# Alfalfa Silage Diet Improves Meat Quality by Remodeling the Intestinal Microbes of Fattening Pigs

**DOI:** 10.3390/foods12173209

**Published:** 2023-08-25

**Authors:** Junying Xu, Xiao Liu, Hongmin Geng, Rui Liu, Fang Li, Jixiang Ma, Mengqi Liu, Boshuai Liu, Hao Sun, Sen Ma, Zhichang Wang, Xiaoyan Zhu, Defeng Li, Chengzhang Wang, Yinghua Shi, Yalei Cui

**Affiliations:** 1 College of Animal Science and Technology, Henan Agricultural University, Zhengzhou 450002, China; ying0508323@163.com (J.X.);; 2 National Engineering Research Center of Wheat and Corn Further Processing, Henan University of Technology, Zhengzhou 450002, China; 3 Henan Key Laboratory of Innovation and Utilization of Grassland Resources, Zhengzhou 450002, China; 4 Henan Forage Engineering Technology Research Center, Zhengzhou 450002, China

**Keywords:** meat quality, alfalfa meal, alfalfa silage, intestinal microbes, fatty acids

## Abstract

Because the demand for pork is increasing, it is crucial to devise efficient and green methods to improve the quality and quantity of meat. This study investigated the improvement in pork quality after the inclusion of alfalfa meal or alfalfa silage in pig diet. Our results indicated that alfalfa silage improved meat quality more effectively in terms of water-holding capacity, drip loss, and marbling score. Besides, an alfalfa silage diet can affect the level of fatty acids and amino acids in pork. Further, alfalfa silage was found to improve meat quality by remodeling intestinal microbiota and altering the level of SCFAs, providing a viable option for improving meat quality through forage.

## 1. Introduction

Pork is an essential part of the human dietary pattern, accounting for a considerable proportion of livestock products. The rise in the human population has led to an increase in the demand for good-quality pork. Such increased demand has resulted in the aggressive selection of fattening pigs with a high growth rate and leanness, ultimately reducing pork quality [1]. Clenbuterol is a kind of growth promoting drug. Adding 3–5 mg/kg of clenbuterol to pig feed can increase lean meat by 9.7% and reduce fat by 14.1%. However, its presence in any form of food is illegal and may have many harmful effects on humans, such as inducing malignant tumors, chromosomal aberrations, metabolic disorders, hypokalemia, and other acute poisonings [2]. According to the declaration of the Ministry of Agriculture of the People’s Republic of China (MAPRC, 2014), China decided to investigate and penalize the illegal inclusion of Clenbuterol in accordance with the law [2]. As a result, improving meat quality safely and effectively to suit peoples’ requirements is one of the most pressing issues in the country [3]. Improving pork quality using feed is regarded as a safe and effective strategy.

Alfalfa, a member of the Medicago genus, is a perennial legume fodder grass renowned as the “king of forage grasses” due to its high protein and fiber content, and widespread cultivation [2,4,5]. Alfalfa, used as a green feed in traditional Chinese pig farming, contains various beneficial compounds, such as saponins, polysaccharides, and flavonoids. Saponins, a class of antioxidants, reduce apoptosis [6,7]. Flavonoids exhibit physiological functions such as anti-inflammatory and anti-tumor cell proliferation [6]. Currently, alfalfa is utilized in livestock and poultry production mainly in the form of green feed, alfalfa silage, and alfalfa meal. Alfalfa meal offers several advantages, such as quick processing, easy transportation, good palatability, a high fiber content, and a high digestibility for animals [8]. Su et al. showed that adding alfalfa meal to the diet of Tibetan sheep appropriately promotes an increase in the content of essential amino acids and flavor amino acids in lamb, and also promotes the deposition of MUFA and PUFA [9]. In another study, adding fresh alfalfa to rabbit feed significantly changed the fat content and fatty acids of the meat, increased the content of ALA, EPA, and DHA, and decreased the proportion of n-6/n-3 [10]. Oppedisano et al. showed that alfalfa meal could increase the growth of beneficial bacteria in sows’ intestines, and improve the reproductive performance and physical condition of sows [11]. Alfalfa silage can alleviate the shortage of green forage in winter and has a relatively long storage time [12]. As well, the nutritional composition of fresh alfalfa and alfalfa silage remains relatively constant. The total concentration of saponins in alfalfa silage increased by two to three times compared to the level in fresh alfalfa [13]. Compared with alfalfa meal, alfalfa silage contains a large number of probiotics and growth promoting factors. In addition, the low pH value of alfalfa silage prevents protein degradation [13]. Research has shown that adding alfalfa silage to broiler feed can have a positive effect on the meat quality [14]. Fermented alfalfa can increase the content of beneficial bacteria and short chain fatty acids in weaned pig colon [15].

Therefore, whether alfalfa, especially alfalfa silage, has beneficial effects on fattening pigs through changing their intestinal microbiota or short-chain fatty acids (SCFAs) has not been investigated. In this study, we investigated the effects of 10% alfalfa meal and 10% alfalfa silage in the normal diet on the growth performance, meat quality, intestinal microbiota, and short-chain fatty acids (SCFAs) of fattening pigs.

## 2. Materials and Methods

### 2.1. Animals and Experimental Design

Two hundred and forty healthy “Duroc ×Landrace × Yorkshire” pigs, each weighing approximately 60 kg, were randomly divided into three groups based on dietary experiment, control group (CON), 10% alfalfa meal group (AM), and 10% alfalfa silage group (AS). Each group comprised four replicates, with 20 pigs in each replicate. The experimental pre-feeding and experimental periods were 7 d and 61 d, respectively. The feeder was a rectangular body made of stainless steel. The feed was manually supplemented. The water dispenser was an automatic water dispenser. The study protocol was approved by the Institutional Animal Ethics Committee of Henan Agricultural University (number, HENAU-2018-039; Zhengzhou, China). Alfalfa silage was obtained from Ping County Minxia Herding Co. (Nanyang, China). The nutritional composition of the alfalfa meal and alfalfa silage is shown in Table 1. The alfalfa silage was a wrapped silage product. The experimental diet (Table 2) was designed in accordance with the nutritional standards of the NRC 2012 in the USA. Pigs had free access to feed and water throughout the study period.

### 2.2. Assessment of Growth Performance

The feed intake of the pigs was monitored, and the average daily feed intake per pig was calculated. Average daily feed intake (ADFI) = average daily supplement (kg) − average daily surplus (kg). The average daily gain (ADG) of pigs was determined by recording the weight of the pigs on the morning of day 8 and day 61. The feed conversion ratio (FCR) was calculated by dividing the average daily feed intake by the average daily weight gain.

### 2.3. Assessment of Pork Quality and Fatty Acid Quantification

We selected 1 pig from each replicate, totaling 12 pigs, taking approximately 500 g of the longest dorsal muscle from each pig for measurement of meat color, marbling, dripping loss, and water holding capacity. The remaining 100 g was used for measurement of fatty acids. The drip loss, water holding capacity, meat color, and marbling pork at 24 h post-slaughter were measured as previously described [16]. The meat color was measured by using a standard colorimetric card. All the evaluations were completed by 10 people, and the evaluators did not know the source of the samples. The rating criteria are gray 1, light gray 2, fresh red 3, slightly deep red 4, and deep red 5. An ion chromatograph (THERMO SCIENTIFIC DIONEX ICS-5000+ SP, Waltham, MA, USA) was used to quantify fatty acids according to GB/T 9695.19.

### 2.4. Amino Acid Profiling

The amino acid level in pig muscles was determined using high-performance liquid chromatography (HPLC). Briefly, 25 mg of muscle samples were freeze-dried in liquid nitrogen and then crushed. The crushed samples were put into 5 mL ampoules. Then, 3 mL hydrochloric acid (6 mol/L) was added to the ampoules. The samples were then hydrolyzed in an oven at 110 °C for 24 h. Then, the samples were washed using a washing solution, transferred to an evaporating dish, and dried at 80 °C. The evaporating dish was washed several times with a derivative buffer solution. The solution was transferred into a 25 mL volumetric flask and fixed and filtered through a 0.45 μm microporous membrane. Then, 160 μL of the solution was put in a test tube. To the test tube, diluted Solution A (0.1 mol/L pH 6.5 sodium acetate solution/ acetonitrile = 93/7) and 100 μL of Solution B (water/acetonitrile = 20/80) were added, shaken well, and incubated for 60 min at room temperature. Then, 400 μL of hexane solution was added and shaken for 5–10 s. The mixture was incubated at room temperature and stratified. Next, the lower 200 μL portion of the solution was removed and mixed with 1000 μL of deionized water. Then, 200 μL of this solution was separated and mixed with 800 μL of deionized water. The mixture was filtered through an organic membrane of 0.22 μm pore size. Then, 10 μL of the filtrate was analyzed using HPLC (LC5090, Zhejiang Fuli Analytical Instruments Co., Ltd., Wenling, China).

### 2.5. Assessment of SCFAs in the Colon

One gram of colonic content was obtained from each pig and diluted in deionized water (mass-to-volume ratio of 1/9). The samples were centrifuged at 10,000 rpm for 10 min. Then, the samples were subjected to ultrasound for 30 min. Then, 1 mL of supernatant was transferred to a 2 mL centrifuge tube. Subsequently, 0.2 mL of 2.5% metaphosphoric acid was added to the supernatant and mixed thoroughly. The solution was incubated at 4 °C for 30 min and then centrifuged at 10,000 rpm for 10 min. Then, 100 μL of supernatant was transferred into another centrifuge tube and mixed with 900 μL of deionized water to dilute the sample 10 times. The diluted solution was filtered through a 0.22 μm microporous membrane. The filtered solution was analyzed using an ion chromatograph to quantify volatile fatty acids.

### 2.6. 16S rRNA Sequencing

The total DNA of the intestinal microbes of colon and cecum were extracted using the E.Z.N.A.^®^ soil DNA kit (Omega Bio-tek, Norcross, GA, USA). The extracted DNA was used as a template for PCR amplification of the V3-V4 variable regions (F-ACTCCTACGGGAGGCAGCAG) and (R-GGACTACHVGGGTWTCTAAT). The amplified sample was sequenced using the Miseq PE300/NovaSeq PE250 platform (Shanghai Meiji Biomedical Technology Co., Ltd., Shanghai, China).

### 2.7. Statistical Analysis

Growth performance, meat quality, and intestinal SCFA levels of fattening pigs were analyzed using SPSS 22.0 software (IBM Corp. Released 2013. IBM SPSS Statistics for Windows, Version 22.0. Armonk, NY, USA: IBM Corp.). Data were evaluated using one-way analysis of variance (ANOVA), and difference between means was assessed using Duncan’s test (*p* ≤ 0.05). All data are expressed as mean ± SD. Differential bacteria with a 95% confidence interval at the microbial level were screened using the Kruskal–Wallis H test and FDR multiple test correction. The marker microbes associated with alfalfa meal and alfalfa silage were further identified using the linear discriminant analysis (LDA) effect size (LEfSe) method. To determine the association of colon microbes with fatty acids, amino acids, and SCFAs, redundancy analysis (RDA) was performed at the genus level using Spearman’s correlation analysis (RDA, 2014). The Benjamini–Hochberg false discovery rate (FDR) correction was used to correct for multiple testing.

## 3. Results

### 3.1. Growth Performance

Table 3 shows the growth performance of fattening pigs from different experimental groups. The AS group exhibited the highest ADG, followed by the AM group and then the CON group. However, there were no significant differences between the ADGs and ADFIs of the three groups. Furthermore, the FCR of the AS group was the least of the three groups. The differences among the FCR of the CON, AM, and AS groups were not significant.

### 3.2. Meat Quality of Fattening Pigs

**Figure 1:**
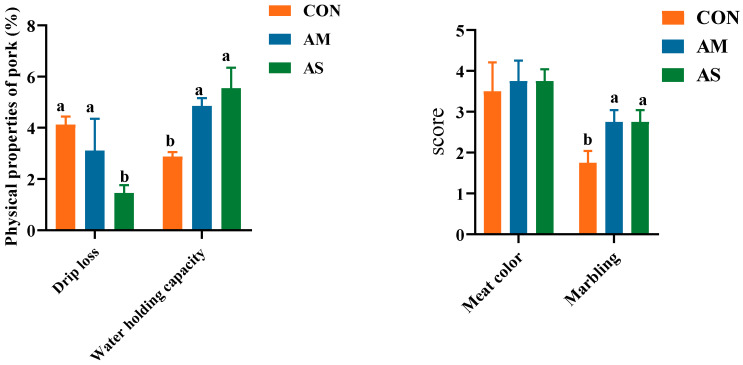
Effects of different diets on the meat quality of fattening pigs. Different letters in the same line indicate significant difference.

The pork quality, including meat color, marbling, drip loss, and water holding capacity, is depicted in Figure 1. The drip loss was significantly lower in the AS group than in the CON and AM groups. The water-holding capacity and marbling score were significantly higher in the AM and AS groups than in the CON group. No significant difference was observed in the meat color of the three groups. The results showed that the AS group exhibited a better drip loss and water-holding capacity than the CON and AM groups.

### 3.3. Muscle Fatty Acid Profile

**Figure 2:**
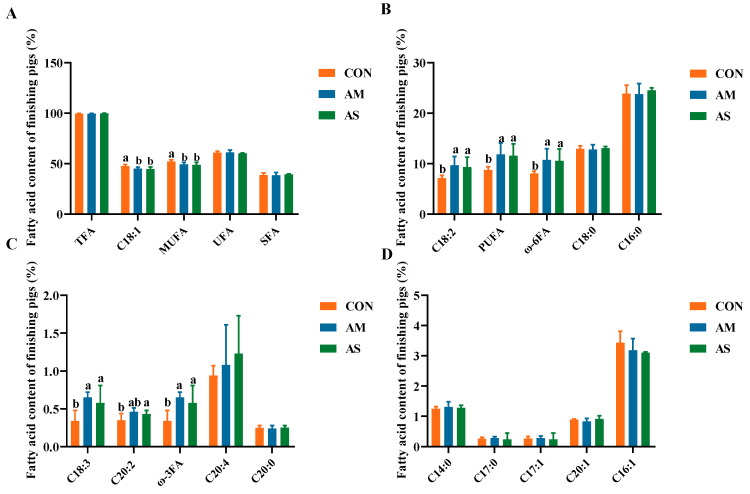
Effects of different diets on the fatty acid content of fattening pigs. (**A**) The content of TFA, C18:1, MUFA, UFA and SFA. (**B**) The content of C18:2, PUFA, ω-6FA, C18:0 and C16:0. (**C**) The content of C18:3, C20:2, ω-3FA, C20:4 and C20:0. (**D**) The content of C14:0, C17:0, C17:1, C20:1 and C16:1. Different letters in the same line indicate significant difference. TFA tallow fatty acid, UFA unsaturated fatty acid, SFA saturated fatty acid.

As shown in Figure 2, the levels of oleic acid (C18:1) and monounsaturated fatty acids (MUFA) were significantly lower in the muscles of the AM and AS groups than in the CON group. The levels of α-linolenic acid (C18:3), linoleic acid (C18:2), polyunsaturated fatty acids (PUFA), eicosadienoic acid (C20:2), ω-3, and ω-6 were significantly higher in the AM and AS groups than in the CON group. The AM and AS groups did not exhibit significant differences in their levels of fatty acids.

### 3.4. Muscle Amino Acid Profile

As shown in Table 4, the AS group exhibited a significantly higher Ser level than the CON group and a significantly lower Phe level than the CON and AM groups. The three groups did not exhibit significant differences in their levels of other amino acids.

### 3.5. Distinct Cecum Microbial Compositions

**Figure 3:**
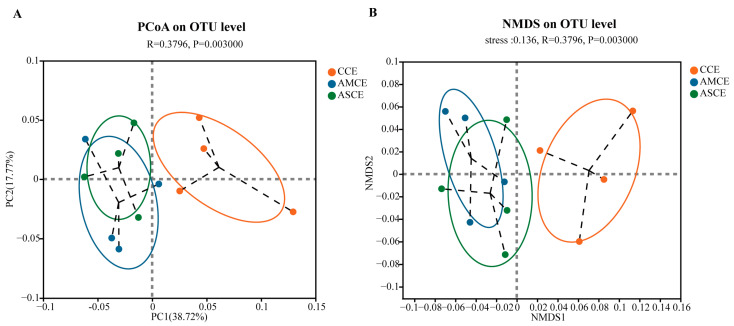
Principal coordinates analysis (PCoA) plot and non-metric multidimensional scaling (NMDS) of the cecum microbiota based on Abund-Jaccard metrics. (**A**) Principal coordinates analysis (PCoA) plot analysis. (**B**) Non-metric multidimensional scaling (NMDS) analysis. CCE, the collection of cecum content of the CON group. AMCE, the collection of cecum content of the AM group. ASCE, the collection of cecum content of the AS group.

**Figure 4:**
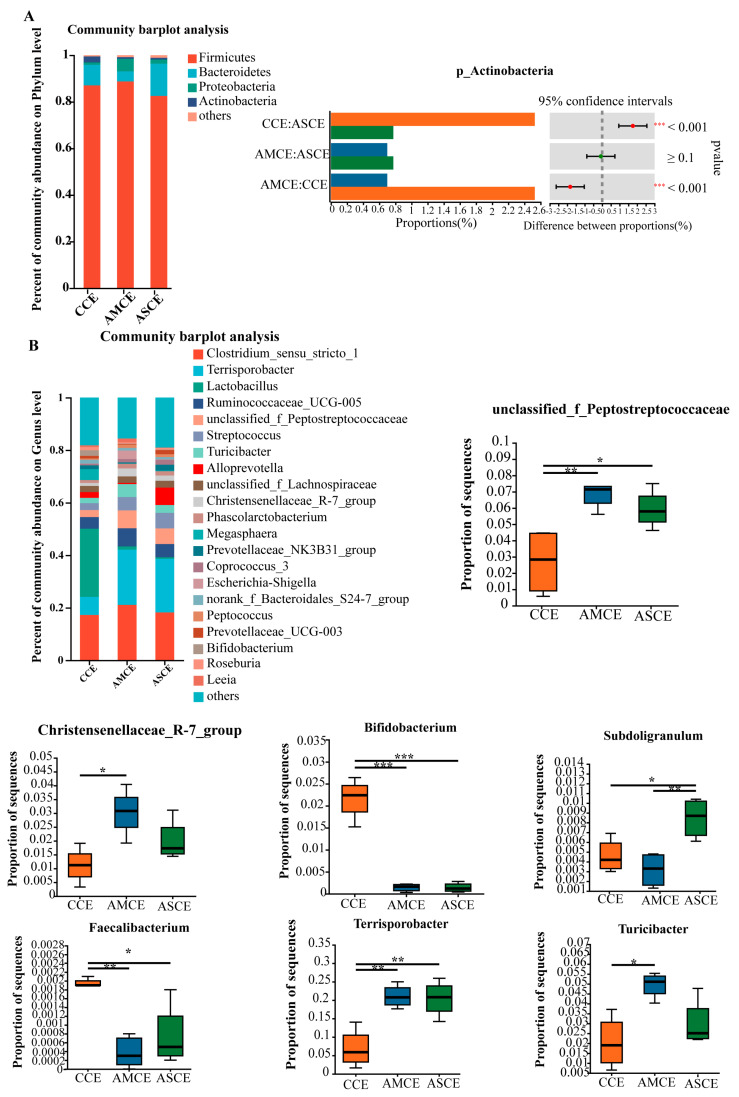
Microbial profile of the cecum. (**A**) Composition of cecum microbiota at the phylum level and analysis of difference. (**B**) Composition of cecum microbiota at the genus level and analysis of difference. * indicates *p* ≤ 0.05, ** indicates *p* ≤ 0.01, *** indicates *p* ≤ 0.001.

PCoA and NMDS analyses (Figure 3) showed that the AMCE (AMCE, the collection of cecum content of the AM group) and ASCE (ASCE, the collection of cecum content of the AS group) were completely separated from the CCE (CCE, the collection of cecum content of the CON group), while most of the AMCE and ASCE overlapped. A statistical analysis of intestinal microbiota (Figure 4A) revealed that, at the phylum level, the cecum microbiota of the three groups primarily comprised Firmicutes, Bacteroidetes, Proteobacteria, and Actinobacteria. More specifically, the microbes in the CCE included Firmicutes (87.13%), Bacteroidetes (8.79%), Actinobacteria (2.49%), and Proteobacteria (1.05%). The microbes in the AMCE included Firmicutes (88.77%), Proteobacteria (5.27%), Bacteroidetes (4.40%), and Actinobacteria (0.70%). The microbes in the ASCE comprised Firmicutes (82.49%), Bacteroidetes (13.80%), Proteobacteria (1.83%), and Actinobacteria (0.77%). These results indicated that the cecum microbiota of all of the experimental groups primarily comprised Firmicutes (>82%). Furthermore, the CCE group harbored a significantly higher level of Actinobacteria than the other two groups.

The cecum microbiota of the three experimental groups exhibited a higher diversity at the genus level (Figure 4B). The most abundant genera in the CCE group included Lactobacillus (25.99%), Clostridium_sensu_stricto_1 (17.35%), and Terrisporobacter (6.84%). In the AMCE group, the most abundant genera comprised Clostridium_sensu_stricto_1 (21.17%), Terrisporobacter (20.80%), and UGG-005 (8.89%). The ASCE group predominantly contained Terrisporobacter (20.38%), Clostridium_sensu_stricto_1 (18.20%), and unclassified_f_Peptostreptococcaceae (5.92%). A differential analysis revealed that the abundances of Terrisporobacter and unclassified_f_Peptostreptococcaceae were significantly higher in the AMCE and ASCE groups than in the CCE group. The abundances of Bifidobacterium and Faecalibacterium in the AMCE and ASCE groups were significantly lower than in the CCE group. The abundances of Turicibacter and Christensenellaceae_R-7_group in the AMCE group were significantly higher than in the CCE group. The abundance of Subdoligranulum was significantly higher than those in the ASCE and CCE groups.

### 3.6. Screening of Biomarkers for Microbes in in the Cecum

**Figure 5:**
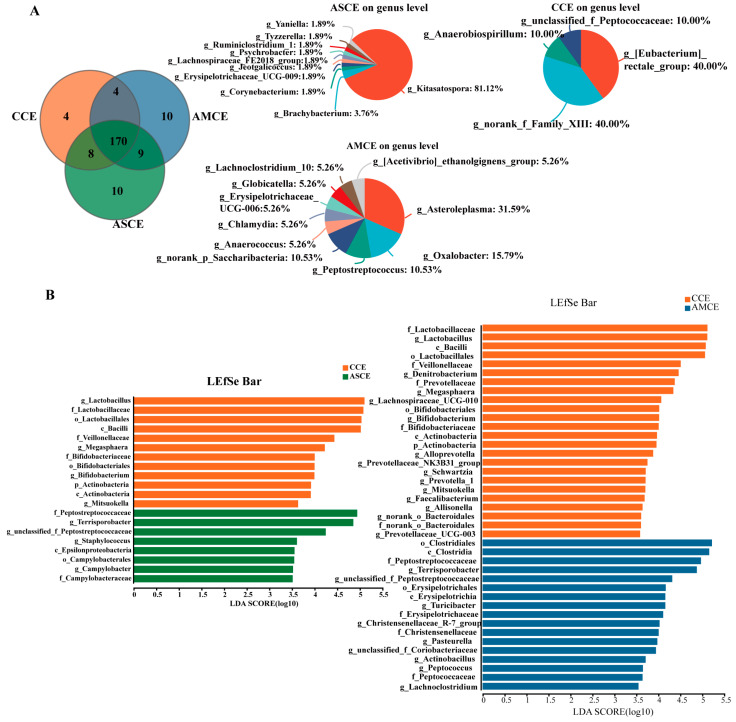
Venn diagram analysis of cecum microbiota and LEfSe analysis in all groups. (**A**) Venn diagram analysis. (**B**) LEfSe analysis.

In the AMCE group, unique microbial genera were detected (Figure 5A), such as g_Asteroleplasma, g_Oxalobacter, g_Peptostreptococcus, g_norank_P_Saccharibacteria, g_Anaerococcusetc, g_Chlamydia, g_Erysipelotrichaceae_UCG-006, g_Clobicatella, g_Lachnoclostridium-10, and the g_[Acetivibrio] ethanolgignens_group. In the ASCE group, g_Kitasatospora and g_Brachybacterium were detected in a relatively high abundance. In the AMCE group, a LEfSe analysis (Figure 5B) revealed the presence of the following markers, g_Terrisporobacter, g_Turicibacter, g_Pasteurella, g_Christensenellaceae_R-7_group, g_Actinobacillus, g_Peptococcus, g_unclassified_f_Peptostreptococcaceae, g_Lachnoclostridium, and g_unclassified_f_Coriobacteriaceae. The markers detected in the ASCE group included g_Terrisporobacter, g_unclassified_f_Peptostreptococcaceae, g_Staphylococcus, and g_Campylobacter.

### 3.7. Distinct Colon Microbial Compositions

**Figure 6:**
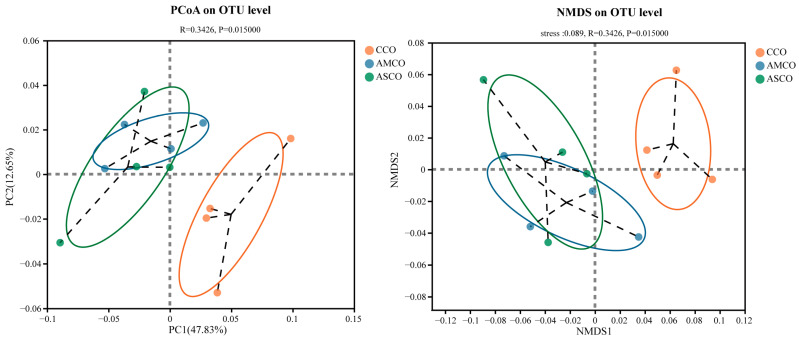
Principal coordinate analysis (PCoA) plot and non-metric multidimensional scaling (NMDS) of the colon microbiota based on Abund-Jaccard metrics.CCO, the collection of colonic content of the CON group. AMCO, the collection of colonic content of the AM group. ASCO, the collection of colonic content of the AS group.

**Figure 7:**
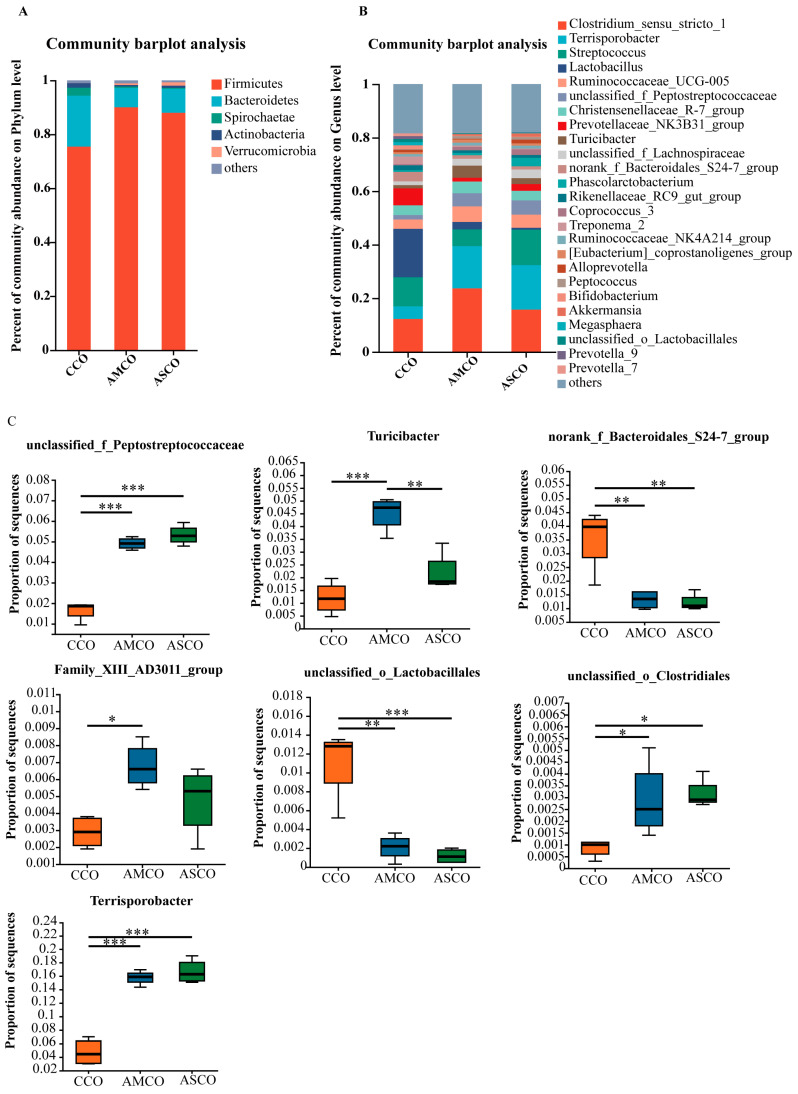
Microbial profile of the colon. (**A**) Composition of colonic microbiota at the phylum level and analysis of difference. (**B**) Composition of colonic microbiota at the genus level. (**C**) Difference analysis of colonic microbiota at the genus level. * indicates *p* ≤ 0.05, ** indicates *p* ≤ 0.01, *** indicates *p* ≤ 0.001. CCO, the collection of colonic content of the CON group. AMCO, the collection of colonic content of the AM group. ASCO, the collection of colonic content of the AS group.

A β-diversity analysis revealed that the microbe composition in the CCO group (CCO, the collection of colonic content of the CON group) was significantly different from those in the AMCO (AMCO, the collection of colonic content of the AM group) and ASCO (ASCO, the collection of colonic content of the AS group) groups (Figure 6). The microbial compositions of the AMCO and ASCO groups did not differ significantly. The microbial compositions of the three groups at the phylum and the genus level were analyzed using the community bar maps. At the phylum level (Figure 7A), the CCO group primarily comprised Firmicutes (75.32%), Bacteroidetes (18.95%), and Spirochaete (2.97%); the AMCO group also primarily comprised Firmicutes (89.98%), Bacteroidetes (7.23%), and Spirochaete (0.58%); and the ASCO group primarily comprised Firmicutes (88.07%), Bacteroidetes (8.79%), and Verrucomicrobia (1.21%). The abundance of Spirochaete was lower in the AMCO (0.58%) and ASCO groups than in the CCO group (2.97%). The three experimental groups did not exhibit any significant difference in the composition of the colonic microbes at the phylum level.

As shown in Figure 7B, at the genus level, the CCO group primarily comprised Lactobacillus (17.96%), Clostridium_sensu_stricto_1 (12.31%), and Streptococcus (10.99%); the AMCO group primarily comprised Clostridium_sensu_stricto_1 (23.58%), Terrisporobacter (15.74%), and Streptococcus (6.27%); and the ASCO group also primarily comprised Clostridium_sensu_stricto_1 (15.64%), Terrisporobacter (16.74%), and Streptococcus (13.20%). A differential analysis revealed that the levels of unclassified_f_Peptostreptococcaceae, Terrisporobacter, and unclassified_o_Clostridiales were significantly higher in the AMCO and ASCO groups than in the CCO group. The levels of norank_f_Bacteroidales_S24-7_group and unclassified_o_Lactobacillales were significantly lower in the AMCO and ASCO groups than in the CCO group. The AMCO group contained significantly more Turicibacter and Family_XIII_ AD3011_group than the CCO group and significantly more Turicibacter than the ASCO group.

### 3.8. Screening of Biomarkers for Microbes in the Colon

**Figure 8:**
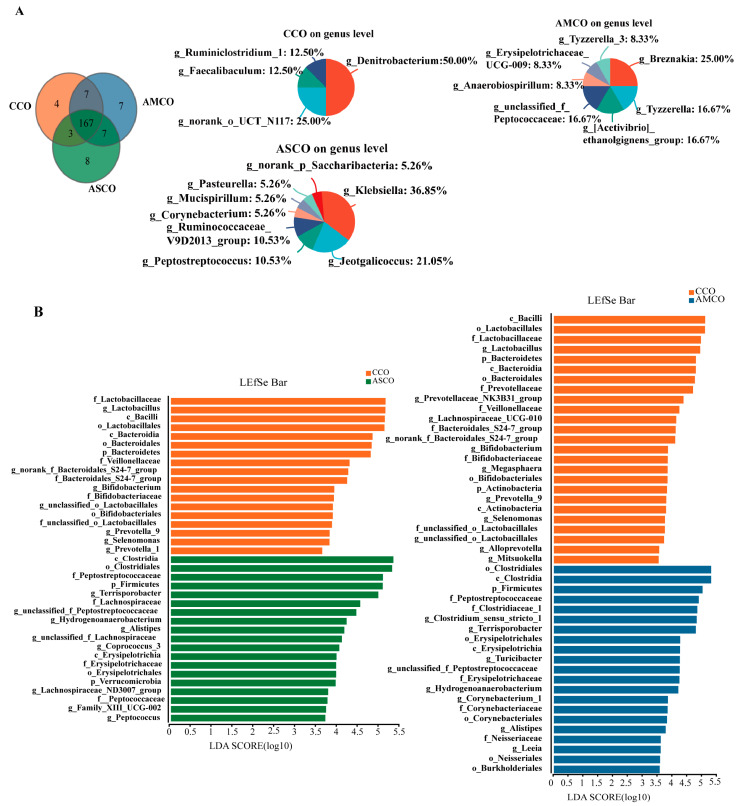
Venn diagram analysis of colonic microbiota profiles and LEfSe analysis in all groups. (**A**) Venn diagram analysis. (**B**) LEfSe analysis. CCO, the collection of colonic content of the CON group. AMCO, the collection of colonic content of the AM group. ASCO, the collection of colonic content of the AS group.

A Venn analysis of colonic microbes (Figure 8A) showed that the CCO, AMCO, and ASCO groups contained four, seven, and eight unique microbial genera, respectively. LEfSe analysis (Figure 8B) revealed the LDA score of Clostridium_sensu_stricto_1 and Terrisporobacter in the AMCO was more than 4.5. In the ASCO, the LDA score of Terrisporobacter was more than 4.5.

### 3.9. Colon SCFAs

**Figure 9:**
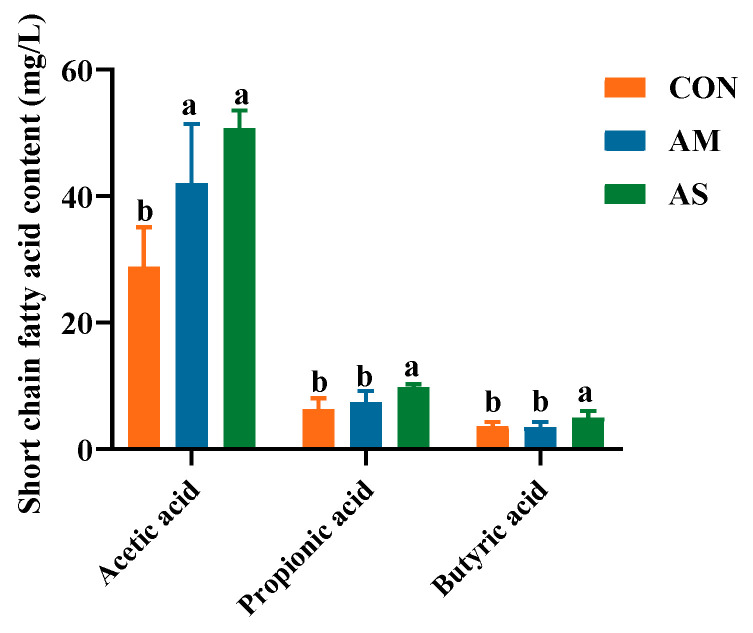
Effects of different experiments on the level of SCFAs in the colon. Different letters in the same line indicate significant difference.

The levels of acetic acid in the AM and AS groups were significantly higher than in the CON group. Furthermore, the levels of propionic acid and butyric acid in the AS groups were higher than in the CON and AS groups (Figure 9).

### 3.10. Correlation Analyses

**Figure 10:**
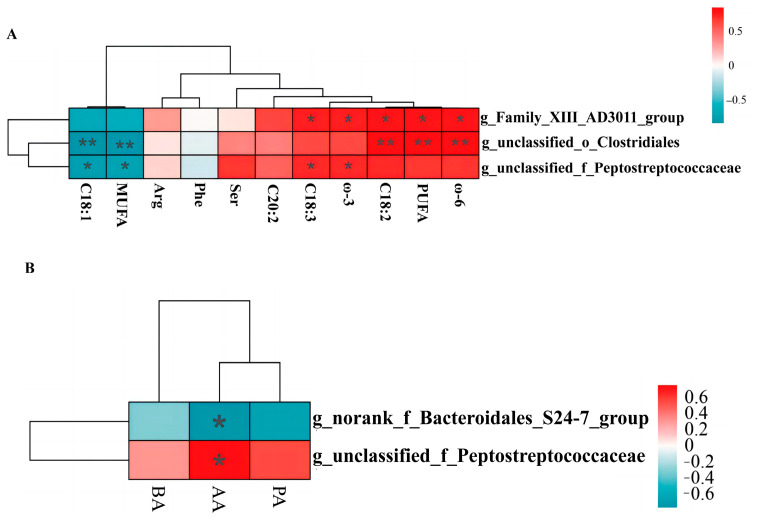
Correlation analysis. (**A**) correlation analysis of muscle fatty acids, amino acids and colonic microbiota. (**B**) correlation analysis of SCFAs and colonic microbiota. BA, Butyric acid. AA, Acetic acid. PA, Propionic acid. * indicates *p* ≤ 0.05, ** indicates *p* ≤ 0.01.

Pearson’s correlation analysis was used to analyze the correlation between microbial markers and the differential levels of SCFAs and fatty acids in the three experimental groups. The results were corrected by FDR. As shown in Figure 10A, g_unclassified_o_Clostridiales and g_Family_AD3011_group were significantly positively correlated with C18:2, PUFA, and ω-6. Further, g_unclassified_o_Clostridiales and g_unclassified_f_Peptostreptococcaceae were negatively correlated with C18:1 and MUFA. In addition, g_Family_XIII_AD3011_group and g_unclassified_f_Peptostreptococcaceae were significantly positively correlated with C18:3 and ω-3.

In terms of SCFAs (Figure 10B), g_norank_f_Bacteroidales_S24-7_group and g_unclassified_f_Peptostreptococcaceae were negatively and positively correlated with acetic acid level, respectively.

## 4. Discussion

### 4.1. Effects of Different Diets on the Growth Performance of Fattening Pigs

To ensure efficient FCR and rapid weight gain of fattening pigs, their diet primarily comprises a mixture of finely processed grains [17]. Previous research has found that a refined, processed, small-particle, and low-fiber diet could lead to mucosal damage in pig stomach [18,19,20]. Alfalfa, the king of forage, is regarded as a green feed in traditional Chinese livestock farming, as it is enriched with insoluble fiber, such as xylan, lignin, and cellulose [21,22]. In the present study, the inclusion of alfalfa meal or alfalfa silage in pig diet led to an increase in the ADG and a decrease in the FCR of both the AM and AS groups compared to the CON group; however, these changes were not significant. Previously, Kass [23] showed that adding 5% alfalfa meal to pig diet did not significantly impact the ADG and FCR of the pigs, which corroborated our results. The mild improvement in the growth performance of pigs in the AS group might be attributed to the consumption of a higher level of nutrients from the alfalfa silage that could be easily digested and absorbed [24].

### 4.2. Effects of Different Diets on the Meat Quality of Fattening Pigs

Pork quality is primarily determined through two aspects. The first aspect is the physical and chemical characteristics of the pork, such as the water-holding capacity, drip loss, meat color, and marbling [25]. The second aspect is the nutrients present in the pork, such as fatty acids and amino acids. Pork quality is influenced by many factors, such as diet, genetics, and environment [26]. Animal feed, undoubtedly, has a direct effect on meat quality. Fermentation is a method of feed processing that helps produce nutrients that are easily absorbed and utilized by animals and produce better economic benefits [27]. Several studies have shown that fermented feeds could improve the meat quality of fattening pigs and enhance the taste of pork [28,29]. The water-holding capacity and drip loss of meat are important properties of fresh meat [30]. Warner noted that drip loss is closely related to meat color, flavor, tenderness, and juiciness [31]. Drip loss prominently impacts meat quality [31]. Meat with a low water-holding capacity and high drip loss rate has a poor flavor after cooking [31]. In the current study, the AM and AS groups exhibited significantly higher water-holding capacities than the CON group. The AS exhibited a significantly lower drip loss than the CON and AM. Thus, in this study, it was found that alfalfa meal and alfalfa silage diet can improve meat quality by significantly increasing the water-holding capacity. In addition, compared to alfalfa meal, alfalfa silage can also improve meat quality by reducing the dripping loss of meat. The marbling was defined and evaluated by the number and spatial distribution of visible white flecks of fat [32]. The appropriate degree of marbling has a beneficial effect on the juiciness, tenderness, palatability, and flavor of meat [33,34,35]. According to the results of the current study, both alfalfa meal and alfalfa silage diet can improve the score of marbling. Because the marbling of meat has a positive effect on meat quality, alfalfa meal and alfalfa silage can improve meat quality by increasing the score of marbling. Simply, alfalfa meal and alfalfa silage can improve meat quality by significantly increasing the water-holding capacity and marbling of meat. Compared with alfalfa meal, alfalfa silage can also reduce the drip loss of meat to improve the quality of meat.

The fatty acids and amino acids in pork prominently affect its quality. We found that the C18:3, C18:2, C20:2, PUFA, ω-3, and ω-6 levels were significantly higher in the AM and AS groups than in the CON group. PUFAs primarily include C18:3, C18:2, ω-3, and ω-6 [36]. PUFA, a member of the fatty acid family, is a critical nutrient for the growth and development of mammals [37,38]. PUFAs benefit human and animal health by regulating signaling pathways [39,40] and inflammatory processes [41]. In this study, the alfalfa silage and alfalfa meal diets enhanced the level of PUFAs in pork. C18:1 is a representative monounsaturated fatty acid (MUFA) that adversely affects endothelial cells and is involved in endothelial cell necrosis, epithelial damage, and neutrophil infiltration [42,43]. In the current study, the pork of the AM and AS groups exhibited significantly lower levels of C18:1 and other MUFAs than the CON group, suggesting that alfalfa meal and alfalfa silage diets could improve meat quality through changing the profile of fatty acids in muscle. Although, the content of soybean oil varied among the three experimental groups in the formula, which may also have an impact on pigs. However, according to the study of Benz, the content of soybean oil had no effect of FCR [44], which is consistent with our findings. Alencar et al. [45] have shown that the addition of soybean oil increased the content of C18:2 and C18:3 in pork. In this study, alfalfa meal and alfalfa silage diets supplied with a much higher amount of soybean oil not only increased the level of C18:2 and C18:3, but also other PUFAs. In general, alfalfa meal and alfalfa silage diets can increase the level of PUFAs and decrease the level of MUFAs to improve the quality of meat, which was attributed to the overall dietary formulation.

In livestock and poultry meat, the amino acids that impact flavor presentation are primarily categorized as flavor amino acids and sweet amino acids. Flavor amino acids mainly include glycine, phenylalanine, glutamic acid, and aspartic acid. Sweet amino acids mainly include serine, threonine, alanine, and proline [46]. In the current study, the inclusion of alfalfa silage in pig diet significantly increased the level of Ser and decreased the level of Phe in pork. The level of amino acid is an important index to evaluate the nutrition of meat [47]. In this study, the pork from the AM and AS groups exhibited altered amino acid profiles. Because alfalfa meal and alfalfa silage diets can increase the amino acid content in meat, the use of alfalfa meal and alfalfa silage diets can be regarded as an effective method to improve the amino acids in meat.

### 4.3. Effects of Different Diets on Gut Microbes and Their Metabolites in Fattening Pigs

A balanced microbe mix in the gastrointestinal tract of animals contributes to the efficient digestion, absorption, and utilization of nutrients [48,49]. In this study, alfalfa meal and alfalfa silage diets could significantly reduce the abundance of the Actinobacteria phylum. The Actinobacteria phylum primarily comprises aerobic bacteria. A previous study indicated that aerobic metabolism produces substances that destroy cellular proteins and decreases the resistance of the body’s cells and tissues to pathogens [50].

In the AMCE groups, g_Terrisporobacter, g_Turicibacter, and g_Christensenellaceae_R-7_group were selected as the markers according to the score of LDA and the abundance. Terrisporobacter is a Gram-positive bacterium that has been shown to ferment glucose, fructose, and cellulose to produce acetic acid [51], and plays an important role in the degradation of organic matter in compost [52,53]. Turicibacter belongs to the Firmicutes [54,55]. It is known to produce C16:0, C18:1, ω-7, and other fatty acids and ferments feed into acetate, butyrate, and lactate [56]. Christensenellaceae_R-7_group, a member of the family Christensenellaceae [57], is associated with host health. Christensenellaceae shows a positive correlation with proteolytic metabolism in animals [58,59]. Diets of alfalfa silage and alfalfa meal could significantly increase the levels of unclassified_f_Peptostreptococcaceae and Terrisporobacter in the cecum of pigs. Alfalfa meal consumption significantly increased the abundance of Turicibacter and Christensenellaceae_R-7_group. The colonic levels of Terrisporobacter, Turicibacter, and unclassified_f_Peptostreptococcaceae were significantly different among the three groups, with matched LDA scores ≥ 3.5. Furthermore, the colonic abundances of g_Terrisporobacter and g_unclassified_f_Peptostreptococcaceae were significantly higher in the AMCO and ASCO groups than in the CCO group. Alfalfa meal consumption increased the abundance of g_Turicibacter. SCFA quantification in the colon revealed significantly higher levels of acetic acid, propionic acid, and butyric acid in the AS group than in the CON group. This finding was attributed to an increase in SCFAs-producing bacteria.

### 4.4. Potential Mechanism to Improve Meat Quality

Meat quality is influenced by several factors, such as breed, diet, pre-slaughter handling, and storage methods. As monogastric animals, pigs are more likely to transfer components from the feed to their muscle and adipose tissue, thus affecting the pork quality [60]. Similarly, the gut microbiota of pigs vary depending on the proportions of protein, fat, and carbohydrates in the feed. This study aimed to determine a potential correlation between gut microbes and meat quality. To this end, the differential microbes in the colon were correlated with fatty acids in muscles, amino acids, and SCFAs in colon content. The results showed a positive correlation between g_unclassified_f_Peptostreptococcaceae and the levels of acetic acid, C18:3, and ω-3. Furthermore, g_unclassified_f_Peptostreptococcaceae showed a significantly negative correlation with C18:1 and MUFA. Previously, Kwan reported that a high abundance of Peptostreptococcaceae is associated with a reduced risk of type II diabetes [61] and changes in ω-3 and its derivative 2-MAG [62]. This study has shown that ω-3 inhibits the growth of inflammation-associated flora in the gut, promotes beneficial gut microbes, and helps to maintain intestinal health [63]. Furthermore, MUFA promotes inflammatory responses in different organs, which can lead to metabolic diseases [64].

The analysis of the association between differential microbes of the colon and fatty acids revealed a significant positive correlation between g_unclassified_f_Peptostreptococcaceae and the level of PUFAs (C18:3 and ω-3). Overall, these results suggest that alfalfa meal or alfalfa silage altered the composition of the gut microbiota, enhanced the abundance of g_unclassified_f_Peptostreptococcaceae, synergistically increased the acetic acid level in the intestine, and promoted the synthesis of fatty acids.

## 5. Conclusions

In conclusion, alfalfa meal and alfalfa silage diets had no significant impact on the growth performance of pigs. However, alfalfa meal and alfalfa silage, especially alfalfa silage, diets can improve pork quality through the aspects of water-holding capacity, drip loss, and marbling score. Besides, alfalfa meal and alfalfa silage diets can affect the level of fatty acids and amino acids in pork. Combing the analysis of correlation, we concluded that alfalfa meal and alfalfa silage diets improved the meat quality of the pork by altering the composition of gut microbiota, synergistically changing the level of SCFAs in the intestine.

## Figures and Tables

**Table 1:** Nutritional composition of the alfalfa meal and alfalfa silage.

	DM (%)	CP (%)	EE (%)	NDF (%)	ADF (%)
Alfalfa silage	23.21	17.38	2.54	36.87	27.55
Alfalfa meal	92.43	15.81	1.79	43.93	30.99

**Table 2:** Diet composition of fattening pigs.

Items	CON	AM	AS	Items	CON	AM	AS
Ingredient				Nutrient composition			
Corn (%)	72.41	65.99	67.18	DE (MJ/kg)	13.67	13.67	13.63
Soya bean meal (%)	18.12	18.03	16.88	CP (%)	15.19	15.77	15.77
Soybean oil (%)	0.00	2.00	2.18	EE (%)	4.47	6.11	6.39
Bran (%)	7.20	2.00	1.72	NDF (%)	12.24	13.15	13.44
Calcium hydrogen phosphate (%)	0.70	0.71	0.71	ADF (%)	5.13	6.78	7.24
Limestone powder (%)	0.36	0.10	0.13	Ca (%)	0.51	0.51	0.54
Alfalfa Meal (%)	0.00	10.00	0.00	P (%)	0.49	0.45	0.45
Alfalfa Silage (%)	0.00	0.00	10.00	AP (%)	0.24	0.25	0.25
1% premix (%)	1.00	1.00	1.00	Lys (%)	0.95	0.95	0.96
Lysine, 98% (%)	0.21	0.17	0.20				
Total (%)	100.00	100.00	100.00				

Note: The 1% premix provided the following per kg of diets, vitamin A 5000 IU, vitamin D3 3000 IU, vitamin E 40.1 IU, vitamin B2 23.2 mg, vitamin B1 20.01 mg, Nicotinic acid 16 mg, Pantothenic acid 10 mg, Biotin 0.168 mg, Folacin 1.28 mg, Cu 11.2 mg, Fe 110 mg, Zn 65.6 mg, Mn 37.6 mg, I 0.47 mg, Se 0.30 mg.

**Table 3:** Effect of the different groups on growth performance of fattening pigs.

Groups	ADG (kg)	ADFI (kg)	FCR
CON	0.85 ± 0.07	2.70 ± 0.02	3.19 ± 0.24
AM	0.88 ± 0.04	2.77 ± 0.16	3.15 ± 0.21
AS	0.92 ± 0.03	2.71 ± 0.15	2.94 ± 0.09

**Table 4:** Effects of different experimental groups on muscle amino acids of fattening pigs.

Items	CON	AM	AS	Items	CON	AM	AS
Asp	2.02 ± 0.04	2.05 ± 0.03	2.02 ± 0.06	leu	1.77 ± 0.03	1.79 ± 0.02	1.77 ± 0.05
Thr	0.94 ± 0.01	0.94 ± 0.02	0.94 ± 0.04	Tyr	0.76 ± 0.02	0.77 ± 0.03	0.75 ± 0.02
Ser	0.74 ± 0.01 b	0.76 ± 0.02 ab	0.79 ± 0.03 a	Phe	1.13 ± 0.03 b	1.17 ± 0.01 a	0.91 ± 0.01 c
Glu	3.38 ± 0.07	3.43 ± 0.04	3.40 ± 0.07	Lys	2.08 ± 0.03	2.11 ± 0.03	2.12 ± 0.06
Gly	0.95 ± 0.05	0.98 ± 0.02	0.95 ± 0.02	His	1.11 ± 0.06	1.16 ± 0.04	1.13 ± 0.06
Ala	1.23 ± 0.03	1.25 ± 0.02	1.23 ± 0.03	Arg	1.50 ± 0.03	1.52 ± 0.01	1.48 ± 0.04
Val	1.14 ± 0.02	1.15 ± 0.02	1.13 ± 0.02	Pro	1.50 ± 0.03	0.82 ± 0.02	0.83 ± 0.02
Met	0.64 ± 0.02	0.63 ± 0.01	0.65 ± 0.04	Trp	0.22 ± 0.01	0.23 ± 0.01	0.24 ± 0.03
ILe	1.05 ± 0.02	1.08 ± 0.01	1.06 ± 0.03				

Note: Different letters in the same line indicate significant difference. Asp (Aspartic acid), Thr (Threonine); Ser (Serine), Glu (Glutamic acid), Gly (Glycine), Ala (Alanine), Val (Valine), Met (Methionine), Ile (Isoleucine), Leu (Leucine), Tyr (Tyrosine), Phe (Phenylalanine), Lys (Lysine), His (Histidine), Arg, (Argnine), Pro, (Proline), Trp (Tryptophane).

## Data Availability

The datasets generated for this study are available on request to the corresponding author.
